# Linking liver fibrosis to cardiac structure, function, and heart disease: a community-based fibrosis-4 index study

**DOI:** 10.3389/fcvm.2026.1777872

**Published:** 2026-10-07

**Authors:** Rongrong Yang, Yubo Li, Xingxu Yan, Jiao Wang, Min Zhu, Sakura Sakakibara, Abigail Dove, Weili Xu

**Affiliations:** 1 Public Health Science and Engineering College, Tianjin University of Traditional Chinese Medicine, Tianjin, China; 2 Aging Research Center, Department of Neurobiology, Care Sciences and Society, Karolinska Institutet, Stockholm, Sweden; 3 National Key Laboratory of Chinese Medicine Modernization, State Key Laboratory of Component-based Chinese Medicine, Tianjin University of Traditional Chinese Medicine, Tianjin, China; 4 Haihe Laboratory of Modern Chinese Medicine, Tianjin, China; 5 Center of Gerontology and Geriatrics, West China Hospital, Sichuan University, Chengdu, China; 6 National Clinical Research Center for Geriatrics, West China Hospital, Sichuan University, Chengdu, China; 7 Department of Neurology, University of Pennsylvania Perelman School of Medicine, Philadelphia, PA, United States

**Keywords:** cardiac phenotypes, community-based cohort study, fibrosis-4 index, heart disease, magnetic resonance imaging

## Abstract

**Background:**

The Fibrosis-4 Index (FIB-4) is a non-invasive tool used to assess advanced liver fibrosis. Although liver fibrosis has been linked to heart disease, its relationship with cardiac structure and function remains unclear.

**Objectives:**

This study examined associations of FIB-4 with incident heart disease and cardiac morpho-functional phenotypes and explored whether cardiac phenotypes statistically accounted for part of the association.

**Methods:**

In this prospective cohort study using the UK Biobank, 416,001 participants without known heart or liver disease at baseline (mean age 56.15 ± 8.09 years; 55.78% female) were followed for up to 17 years. FIB-4, calculated from age, AST, ALT, and platelet count, was analyzed continuously and using a high-risk threshold of >2.67. Incident coronary heart disease, cardiac arrhythmias (CA), and heart failure (HF) were identified from linked records. A subset of 33,927 participants underwent cardiovascular magnetic resonance (CMR) imaging a median of 10.20 years after baseline.

**Results:**

Over a median follow-up of 14.61 years, 57,207 participants (13.75%) developed heart disease. Higher FIB-4 was associated with CA (HR = 1.32, 95% CI 1.24–1.41), HF (HR = 1.29, 95% CI 1.14–1.47), greater LV mass (*β*=0.014, 95% CI 0.0003–0.027), chamber dilation, reduced atrial ejection fractions, and LV remodeling. Exploratory pathway analysis indicated that LA ejection fraction statistically accounted for 36.38% of the FIB-4–CA/HF association; causal mediation cannot be inferred.

**Conclusion:**

Elevated FIB-4 was associated with incident CA and HF and with adverse CMR phenotypes. Exploratory pathway results were consistent with indirect pathways through cardiac remodeling, but the observational design and single CMR assessment preclude causal or temporal interpretation. FIB-4 may help identify people at higher cardiovascular risk.

## Background

Liver fibrosis, characterized by the excessive accumulation of connective tissue, is a key driver in the progression of chronic liver disease, potentially leading to cirrhosis, liver failure, and hepatocellular carcinoma (1–3). With over 0.27 billion people affected globally, liver fibrosis significantly contributes to the burden of liver disease, increasing healthcare costs and mortality rates (4). The fibrosis-4 index (FIB-4) is a widely used, validated non-invasive marker for detecting liver fibrosis across multiple etiologies and serves as a first-line tool for risk stratification in clinical practice (5, 6). It is calculated using readily available clinical parameters, making it both cost-effective and easy to implement in routine care.

Common types of heart disease include coronary heart disease, cardiac arrhythmias (CA) and heart failure (HF) (7). Although several population-based cohort studies have investigated the relationship between FIB-4 and heart disease, their findings have been inconsistent. Some studies showed an increased risk of heart disease among people with higher FIB-4 (8–12), but others indicated no clear association (13, 14). Furthermore, longitudinal evidence on the association between FIB-4 and different subtypes of heart disease remain unclear. Specifically, only one study explored the association between FIB-4 and atrial fibrillation and found no association (15). Therefore, the association between FIB-4 and heart disease subtypes requires further investigation.

Quantitative assessment of cardiac morphology and function using cardiovascular magnetic resonance (CMR) is critical for identifying pathological conditions in heart disease (16). Growing evidence suggests that increased left ventricular (LV) mass, enlarged ventricular and atrial volumes, reduced systolic function, and abnormal LV remodeling are associated with adverse cardiovascular outcomes (17–19). However, population-based evidence on associations of FIB-4 with cardiac structure and function is limited. It is also unclear whether adverse cardiac phenotypes statistically account for part of the association between FIB-4 and incident heart disease.

In this community-based longitudinal UK Biobank study, we aimed to: (1) examine associations of FIB-4 with incident heart disease and major subtypes, (2) investigate associations of FIB-4 with cardiac structure and function, and (3) explore cardiac phenotypes as potential statistical indirect pathways linking FIB-4 with CA/HF. The third aim was hypothesis-generating and was not intended to establish causal mediation. We hypothesized that higher FIB-4 would be associated with greater risks of incident CA and HF and with adverse cardiac structural and functional phenotypes.

## Methods

### Study design, settings, and participants

The UK Biobank is a large prospective, nationwide cohort study including over 500,000 individuals between the ages of 40 and 70 from across the United Kingdom (20). The baseline examination took place between 2006 and 2010, and involved collection of physical measures, biological samples, and information on health status and sociodemographic and lifestyle factors. Between May 2014 and March 2019, a subset of approximately 50,000 participants underwent imaging assessments, including the collection of structural and functional cardiac parameters from CMR. Changes in health status were monitored via linkage with medical records. This study was reported in accordance with the Strengthening the Reporting of Observational Studies in Epidemiology (STROBE) statement (21).

Of 502,412 total participants, we excluded individuals with baseline heart disease (*n* = 33,772), severe thrombocytopenia (*n* = 290), baseline liver disease (*n* = 4,563), or acute hepatitis (*n* = 119). An additional 46,613 individuals were excluded because of missing FIB-4 data, and 1,054 were lost to follow-up. Ultimately, 416,001 participants were included in analyses of FIB-4 and incident heart disease. Of these, 33,927 participants with CMR measurements were included in analyses of FIB-4 and cardiac morphological and functional parameters (Figure 1).

**Figure 1:**
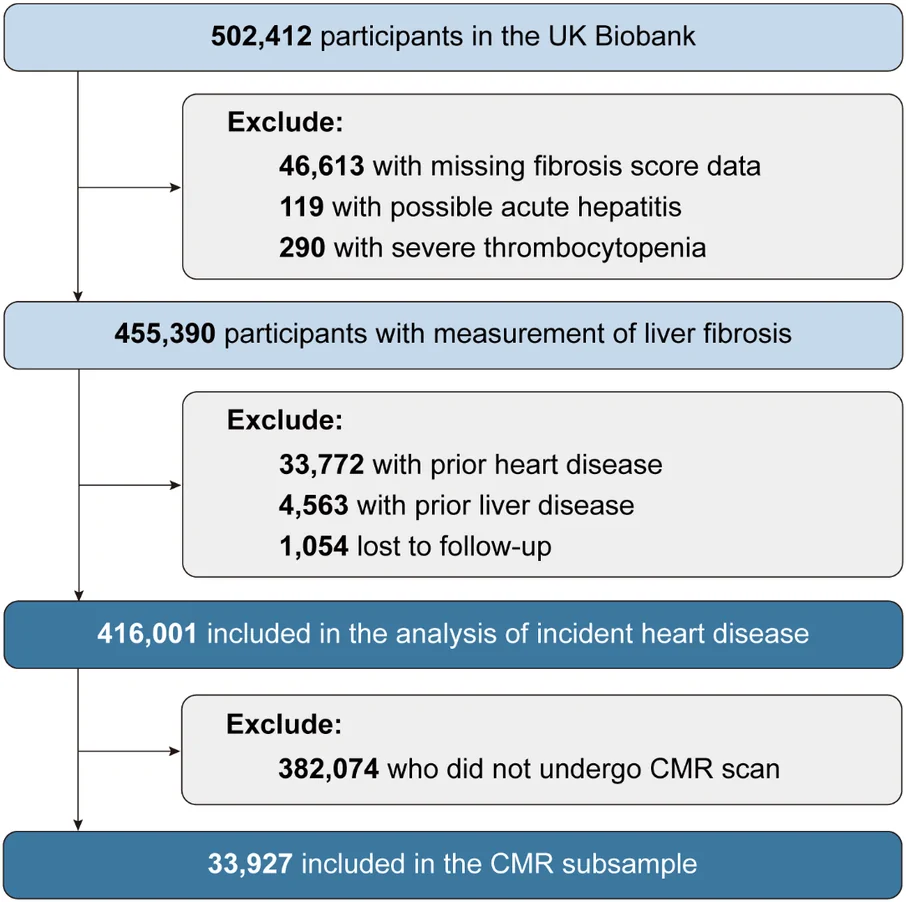
Study population flow chart.

### Data collection

Information on sociodemographic factors (including sex, age, race/ethnicity, education, and socioeconomic status) and lifestyle factors (including smoking status, alcohol consumption status, and physical activity) was self-reported at baseline via touch-screen-based questionnaires. Height and weight were measured and used to calculate body mass index (BMI) [weight in kilograms/(height in meters)^2^] and body surface area (BSA) [(weight in kilograms   ×   height in centimeters)/3600]^½^ (22). Race/ethnicity was categorized as white (including British, Irish, and any other white background) or non-white (including Asian, Black, mixed race, or other background). Education was dichotomized according to whether participants had attained a college/university degree. Socioeconomic status was assessed using the Townsend Deprivation Index, a measure of neighborhood-level socioeconomic deprivation. Smoking and alcohol consumption were categorized as never, previous, or current. Regular physical activity was defined as ≥150 minutes per week of moderate activity or ≥75 min per week of vigorous activity, as measured using the International Physical Activity Questionnaire (23).

Type 2 diabetes was defined as glycated hemoglobin A1c ≥ 6.5%, fasting plasma glucose ≥126 mg/dL, random blood glucose ≥200 mg/dL, use of diabetes medications, medical record of diabetes (International Classification of Diseases, 10th edition [ICD-10] codes: E10-E14), or self-reported history of diabetes (24). Hypertension was defined as systolic blood pressure (SBP) > 130 mmHg, diastolic blood pressure (DBP) > 80 mmHg, use of antihypertensive medications, medical records (ICD-10 codes: I10-I13, I15), or self-reported history of hypertension (25). High cholesterol was defined based on total cholesterol >271 mg/dL, use of cholesterol-lowing medications, medical records (ICD-10 codes: E78), or self-reported history of high cholesterol (26). Metabolic syndrome was diagnosed if participants had at least three of the following five criteria: waist circumference >102 cm in men or >88 cm in women, triglycerides >150 mg/dL, high density lipoprotein cholesterol <40 mg/dL in men or <50 mg/dL in women, SBP >130 mmHg or DBP >85 mmHg, and serum glucose >100 mg/dL (27). Information on liver disease (including liver cancer, chronic viral hepatitis, severe liver disease, or other causes of liver disease) was obtained from medical records and self-reported medical history (28).

### Assessment of FIB-4

FIB-4 was calculated from age, platelet count, aspartate aminotransferase (AST), and alanine aminotransferase (ALT) according to the following formula (6):

$$FIB-4=\frac{\mathrm{Age}(\mathrm{years})\times \mathrm{AST}(U/L)}{\mathrm{Platelet}\;\mathrm{count}(10^{9}/L)\times \sqrt{\mathrm{ALT}(U/L)}}$$

FIB-4 was analyzed both continuously and categorically. We selected the established specificity-oriented threshold of >2.67 *a priori* to identify participants at high risk of advanced liver fibrosis and to facilitate comparison with previous population studies; participants with FIB-4 ≤ 2.67 formed the low/indeterminate-risk group (27, 29). Because age is a component of FIB-4 and a fixed threshold may perform differently across age groups, age-specific thresholds were evaluated in sensitivity analyses. Throughout the revised manuscript, the categorical exposure is described as high versus low/indeterminate FIB-4 risk rather than biopsy-confirmed liver fibrosis.

### Assessment of heart disease

Prevalent and incident cases of heart disease were identified based on medical records and death registry records (ICD-10 codes: I20–I25 and I48–I50) and self-reported history of heart disease. The major subtypes of heart disease included: (1) coronary heart disease (including angina pectoris (I20), myocardial infarction (I21–I23), other acute ischemic heart diseases (I24), and chronic ischemic heart disease (I25)); (2) CA (including atrial fibrillation and flutter (I48), and other CA (I49)); and (3) HF (I50).

### Assessment of cardiac structure and function

Quantitative phenotypes reflecting cardiac structure and function were derived from CMR images. The full UK Biobank CMR protocol has been described previously (16). In brief, CMR images were obtained using clinical wide-bore 1.5-T scanners (MAGNETOM Aera, Syngo Platform VD13A, Siemens Healthcare, Erlangen, Germany). Cardiac chamber parameters in short- and long-axis views were measured using cvi42 post-processing software (Version 5.1.1, Circle Cardiovascular Imaging Inc., Calgary, Canada). The phenotypes included LV end-diastolic volume (EDV), end-systolic volume (ESV), stroke volume (SV), ejection fraction (EF), mass, and geometry; corresponding RV volumes and EF; and maximum volume, minimum volume, SV, and EF for both atria. LV geometry was classified using sex-specific upper reference limits for LVMI (>72 g/m^2^ in men and >55 g/m^2^ in women) and LVMVR (>0.84 g/mL in men and >0.81 g/mL in women): normal geometry (normal LVMI and normal LVMVR), concentric remodeling (normal LVMI and increased LVMVR), eccentric hypertrophy (increased LVMI and normal LVMVR), and concentric hypertrophy (increased LVMI and increased LVMVR) (30).

### Statistical analysis

Baseline characteristics of participants by fibrosis status were compared using *χ^2^* tests for categorical variables, *t* tests for continuous variables with a normal distribution, and Wilcoxon rank-sum tests for continuous variables with a non-normal distribution. Baseline characteristics are presented as mean (standard deviation [SD]) for normally distributed continuous variables, median (interquartile range [IQR]) for non-normally distributed continuous variables, and frequency (percentage) for categorical variables (31).

Associations of FIB-4 with incident heart disease and its subtypes were estimated using Cox proportional hazards regression. Age was used as the underlying timescale, from age at baseline until age at diagnosis, death, or November 15, 2023, whichever occurred first; this provides fine control for age in the time-to-event models. Associations with LV geometric patterns were assessed using Cox models from baseline to the imaging visit. Associations with continuous CMR phenotypes were estimated using multivariable linear regression, with age included as a covariate. The 20th percentile differences (PDs) and 95% CIs in time to heart disease were estimated using Laplace regression.

For all analyses, FIB-4 was first treated categorically and then continuously per 1-unit increment. Models were adjusted for age (where age was not the timescale), sex, BMI, race/ethnicity, education, socioeconomic deprivation, smoking, alcohol consumption, physical activity, hypertension, diabetes, high cholesterol, and metabolic syndrome. In sensitivity analysis, additional models further adjusted for baseline statin use and angiotensin-converting enzyme inhibitor/angiotensin receptor blocker (ACE inhibitor/ARB) use. SGLT2 inhibitor use was not included because UK Biobank baseline recruitment ended before this drug class entered routine clinical use.

Exploratory mediation analysis used generalized structural equation modeling to quantify indirect statistical pathways from baseline FIB-4 to the composite CA/HF outcome through CMR phenotypes. Bias-corrected 95% CIs for indirect effects were estimated using 5,000 bootstrap iterations. Because CMR was measured once and the temporal order between individual CMR phenotypes and subsequent events could not be established for every participant, these analyses were interpreted as associational and not as evidence of causal mediation.

Missing values for ethnicity (*n* = 1,900), education (*n* = 4,767), Townsend deprivation index (*n* = 521), smoking (*n* = 2,003), alcohol consumption (*n* = 985), physical activity (*n* = 33,475), and BMI (*n* = 1,538) were imputed using fully conditional specification, with estimates pooled across five imputed datasets. Sensitivity analyses repeated the main models after complete-case restriction and after defining possible advanced fibrosis using age-specific FIB-4 thresholds (>2.0 for participants aged ≥65 years and >1.3 for those aged 35–65 years) (32) or the nonalcoholic fatty liver disease fibrosis score (NFS >0.675) (5). The primary FIB-4 model was additionally stratified by age (<65 and ≥65 years), with multiplicative interaction tested, and the clinical and CMR models were repeated after adding baseline statin and ACE inhibitor/ARB indicators (Supplementary Tables S10–S12). No formal sample-size calculation was performed; the sample size was determined by the number of eligible UK Biobank participants with available exposure and outcome data. No formal adjustment for multiple comparisons was applied because the CMR and pathway analyses were exploratory. All analyses were conducted in Stata 17.0 (StataCorp LP, College Station, TX); two-sided *P* < 0.05 denoted statistical significance.

## Results

### Baseline characteristics of the study population

Among all 416,001 participants included in this study, 232,048 (55.78%) were women, and the mean (SD) age was 56.15 (8.09) years. Baseline characteristics of the study population by FIB-4 risk are presented in Table 1. A total of 7,735 participants (1.86%) had high-risk FIB-4. Compared to those with low/indeterminate-risk FIB-4, participants with high-risk FIB-4 were older, more likely to be male, and had higher prevalence of hypertension, diabetes, high cholesterol, and metabolic syndrome.

**Table 1:** Baseline characteristics of the study participants.

Characteristics	High-risk FIB-4 (>2.67) (*N* = 7,735)	Low/indeterminate-risk FIB-4 (≤2.67) (*N* = 408,266)	*P* value*
Age, years, mean (SD)	62.23 (6.08)	56.03 (8.08)	< 0.001
Female, *n* (%)	2,860 (36.97)	229,188 (56.14)	< 0.001
White ethnicity, *n* (%)	7,014 (90.68)	371,620 (91.02)	0.297
Education (College), *n* (%)	2,356 (30.46)	136,489 (33.43)	< 0.001
Regular physical activity, *n* (%)	5,785 (74.79)	294,975 (72.25)	< 0.001
Townsend deprivation index, mean (SD)	−1.20 (3.17)	−1.36 (3.05)	< 0.001
Body mass index, kg/m^2^, mean (SD)	27.07 (4.78)	27.28 (4.72)	< 0.001
Smoking status, *n* (%)			< 0.001
Never	3,960 (51.20)	228,203 (55.90)	
Previous smoker	3,054 (39.48)	137,402 (33.66)	
Current smoker	721 (9.32)	42,661 (10.45)	
Alcohol consumption status, *n* (%)			0.016
Never	365 (4.72)	17,459 (4.28)	
Previous drinking	288 (3.72)	13,454 (3.30)	
Current drinking	7,082 (91.56)	377,353 (92.43)	
Hypertension, *n* (%)	5,088 (65.78)	212,356 (52.01)	< 0.001
Diabetes, *n* (%)	591 (7.64)	17,892 (4.38)	< 0.001
High cholesterol, *n* (%)	2,589 (33.47)	113,278 (27.75)	< 0.001
Metabolic syndrome, *n* (%)	2,214 (28.62)	109,906 (26.92)	0.001
AST, U/L, median (IQR)	32.90 (26.80, 48.10)	24.10 (20.90, 28.40)	< 0.001
ALT, U/L, median (IQR)	20.99 (14.75, 32.95)	19.92 (15.29, 27.03)	< 0.001
Platelet count, 10^9^ cells/L, mean (SD)	156.46 (46.07)	256.36 (57.94)	< 0.001
FIB-4 score, mean (SD)	3.40 (1.05)	1.27 (0.42)	< 0.001

*P value was calculated by comparing baseline characteristics between participants with high-risk FIB-4 (>2.67) and those with low/indeterminate-risk FIB-4 (≤2.67).

FIB-4, Fibrosis-4 score; AST, Aspartate aminotransferase; ALT, Alanine aminotransferase; IQR, interquartile range; SD, standard deviation.

### Association between FIB-4 and heart disease

Over a median follow-up of 14.61 years (interquartile range: 13.71–15.36 years), 57,207 participants (13.75%) developed heart disease. In multi-adjusted Cox regression models, high-risk FIB-4 was associated with an increased risk of heart disease (HR = 1.18, 95% CI 1.12–1.23). Laplace regression analysis indicated that participants with high-risk FIB-4 developed heart disease 1.71 years earlier (20th PD; 95% CI −2.14 to −1.29) than those with low/indeterminate-risk FIB-4.

Regarding heart disease subtypes, high-risk FIB-4 was significantly associated with HF (HR = 1.29, 95% CI 1.14–1.47) and CA (HR = 1.32, 95% CI 1.24–1.41), including atrial fibrillation and flutter (HR = 1.33, 95% CI 1.24–1.43) and other forms of CA (HR = 1.26, 95% CI 1.06–1.49). Laplace regression analysis further showed that high-risk FIB-4 anticipated the onset of CA by 2.38 years (20th PDs; 95% CI −2.92 to −1.84) and HF by 1.67 years (20th PDs; 95% CI −2.48 to −0.86) compared to participants with low/indeterminate-risk FIB-4 (Table 2, Figure 2, and Supplementary Table S1).

**Table 2:** Multi-adjusted hazard ratios (HRs), 20th percentile differences (PDs, years), and 95% confidence intervals (95% CIs) for association between the fibrosis-4 index (FIB-4) and different forms of heart disease.

Heart disease	No. of case	Primary approach: High-risk versus low/indeterminate-risk FIB-4		Secondary approach: For each 1.0 unit increase in FIB-4	
		Multi-adjusted HR (95% CI)^a^	Multi-adjusted 20th PDs (95% CI)^a^	Multi-adjusted HR (95% CI)^a^	Multi-adjusted 20th PDs (95% CI)^a^
All-cause heart disease	57,207	1.18 (1.12, 1.23)	−1.71 (−2.14, −1.29)	1.07 (1.06, 1.09)	−0.73 (−0.85, −0.62)
Coronary heart disease	29,785	1.02 (0.95, 1.10)	−0.52 (−1.12, 0.09)	0.98 (0.96, 1.005)	−0.03 (−0.24, 0.18)
Angina pectoris	9,246	0.96 (0.84, 1.10)	0.15 (−0.90, 1.21)	1.04 (0.996, 1.08)	−0.42 (−0.73, −0.11)
Myocardial infarction	8,523	1.01 (0.88, 1.17)	−0.23 (−1.25, 0.79)	0.99 (0.95, 1.04)	−0.05 (−0.35, 0.25)
Other acute ischaemic heart diseases	1,290	1.12 (0.80, 1.57)	−0.82 (−2.66, 1.02)	1.12 (1.01, 1.23)	−0.73 (−1.29, −0.17)
Chronic ischaemic heart disease	19,776	1.02 (0.93, 1.10)	−0.32 (−0.99, 0.35)	0.98 (0.95, 1.01)	−0.01 (−0.23, 0.21)
Cardiac arrhythmias	25,651	1.32 (1.24, 1.41)	−2.38 (−2.92, −1.84)	1.17 (1.14, 1.19)	−1.34 (−1.52, −1.15)
Atrial fibrillation and flutter	21,360	1.33 (1.24, 1.43)	−2.40 (−2.98, −1.82)	1.17 (1.15, 1.20)	−1.33 (−1.49, −1.18)
Other cardiac arrhythmias	4,663	1.26 (1.06, 1.49)	−1.75 (−2.95, −0.55)	1.12 (1.06, 1.18)	−0.90 (−1.21, −0.58)
Heart failure	6,586	1.29 (1.14, 1.47)	−1.67 (−2.48, −0.86)	1.13 (1.08, 1.17)	−0.82 (−1.08, −0.55)

a Adjusted for age, sex, body mass index, ethnicity, physical activity, Townsend deprivation index, education, smoking status, alcohol consumption status, hypertension, diabetes, high cholesterol, and metabolic syndrome.

**Figure 2:**
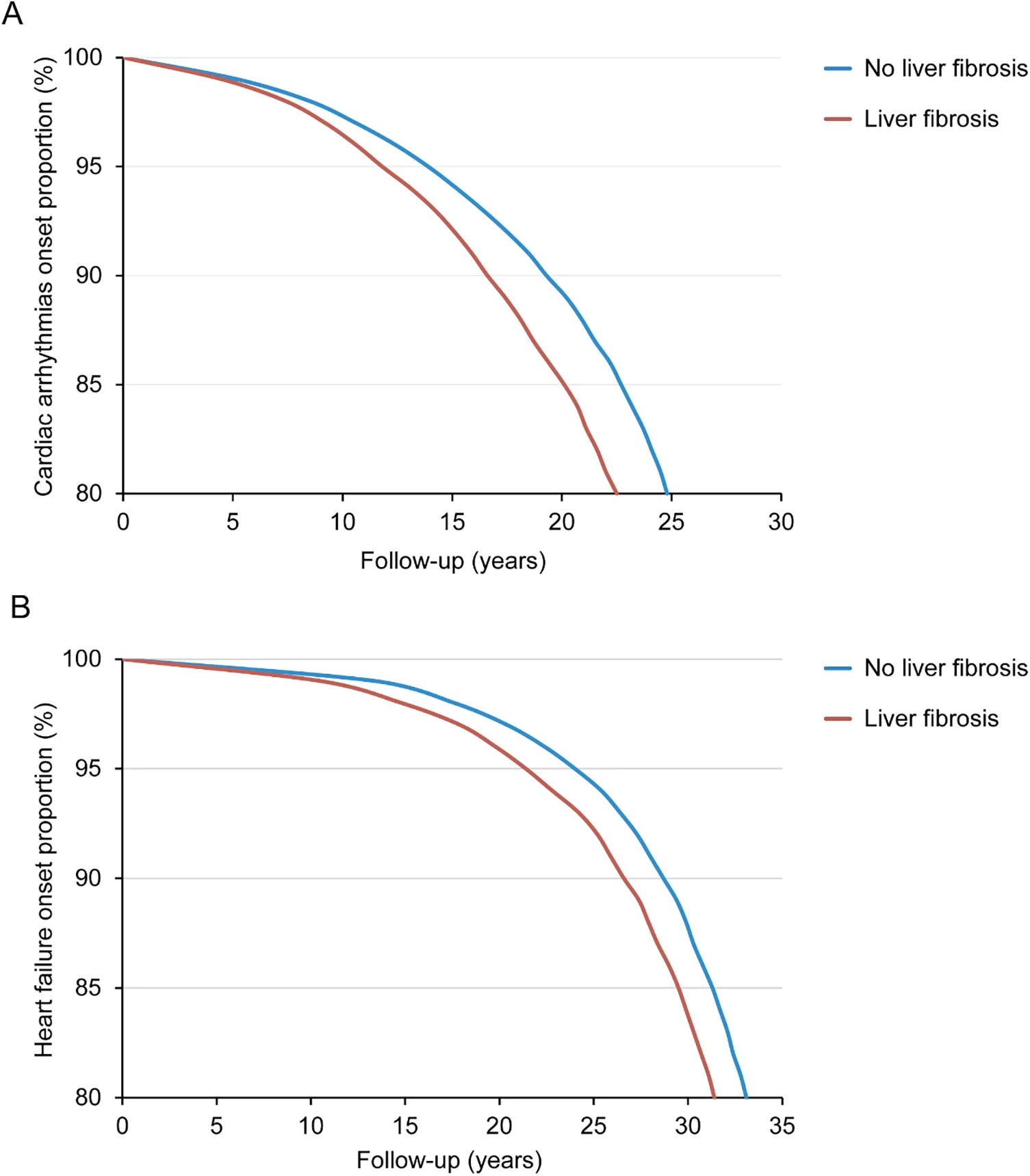
Laplace regression analysis of 20th-percentile differences in time to heart disease onset for high-risk versus low/indeterminate-risk FIB-4. **(A)** Time to incident cardiac arrhythmias; **(B)** time to incident heart failure. Models adjusted for age, sex, BMI, ethnicity, education, Townsend deprivation index, smoking, alcohol consumption, physical activity, hypertension, diabetes, high cholesterol, and metabolic syndrome.

Similar results were observed when FIB-4 score was treated as a continuous variable. Higher FIB-4 scores were associated with an increased risk of heart disease (HR = 1.07, 95% CI 1.06–1.09), HF (HR = 1.13, 95% CI 1.08–1.17) and CA (HR = 1.17, 95% CI 1.14–1.19), including atrial fibrillation and flutter (HR = 1.17, 95% CI 1.15–1.20) and other CA (HR = 1.12, 95% CI 1.06–1.18) (Table 2 and Supplementary Table S1).

### Association between FIB-4 and cardiac phenotypes

Over a median follow-up of 10.20 years (interquartile range: 9.42–10.80 years), 33,927 participants underwent CMR. In multi-adjusted linear regression models, individuals with high-risk FIB-4 showed significantly higher LV volume (LV EDV: *β*=0.033, 95% CI 0.018–0.047; LV ESV: *β*=0.044, 95% CI 0.023–0.064; LV SV: *β*=0.024, 95% CI 0.008–0.040), LV mass (*β*=0.014, 95% CI 0.0003–0.027), RV volume (RV EDV: *β*=0.025, 95% CI 0.010–0.039; RV ESV: *β*=0.022, 95% CI 0.003–0.042; RV SV: *β*=0.026, 95% CI 0.009–0.043), and LA volume (LA maximal volume: *β*=0.051, 95% CI 0.025–0.077; LA minimal volume: *β*=0.083, 95% CI 0.039–0.128; LA SV: *β*=0.038, 95% CI 0.016–0.060). High-risk FIB-4 was also associated with lower LA EF (*β*=-0.899, 95% CI −1.690–−0.108). Additionally, individuals with high-risk FIB-4 had a higher risk of concentric remodeling (HR = 2.12, 95% CI 1.15–3.91). No significant associations were found between high-risk FIB-4 and other cardiac or LV geometric remodeling patterns (Table 3 and Supplementary Table S2).

**Table 3:** Multi-adjusted *β* coefficients and 95% confidence intervals (CIs) for the association between the fibrosis-4 index (FIB-4) and cardiac phenotypes.

Phenotype	No. of subject	Primary approach: High-risk versus low/indeterminate-risk FIB-4	Secondary approach: For each 1.0 unit increase in FIB-4
		Multi-adjusted *β* (95% CI)^a^	Multi-adjusted *β* (95% CI)^a^
LV EDV^b^	33,624	0.033 (0.018, 0.047)	0.033 (0.029, 0.037)
LV ESV^b^	33,624	0.044 (0.023, 0.064)	0.040 (0.034, 0.046)
LV SV^b^	33,624	0.024 (0.008, 0.040)	0.028 (0.024, 0.033)
LV mass^b^	33,626	0.014 (0.0003, 0.027)	0.017 (0.013, 0.020)
LV EF	33,624	−0.473 (−0.975, 0.028)	−0.276 (−0.420, −0.131)
RV EDV^b^	33,624	0.025 (0.010, 0.039)	0.033 (0.029, 0.037)
RV ESV^b^	33,624	0.022 (0.003, 0.042)	0.036 (0.030, 0.042)
RV SV^b^	33,624	0.026 (0.009, 0.043)	0.031 (0.026, 0.036)
RV EF	33,624	0.093 (−0.406, 0.593)	−0.131 (−0.275, 0.014)
LA maximal volume^b^	33,272	0.051 (0.025, 0.077)	0.044 (0.036, 0.051)
LA minimal volume^b^	33,272	0.083 (0.039, 0.128)	0.069 (0.056, 0.082)
LA SV^b^	33,272	0.038 (0.016, 0.060)	0.030 (0.023, 0.036)
LA EF	33,272	−0.899 (−1.690, −0.108)	−0.828 (−1.057, −0.599)
RA maximal volume^b^	33,272	0.021 (−0.003, 0.046)	0.046 (0.039, 0.053)
RA minimal volume^b^	33,272	0.031 (−0.002, 0.064)	0.063 (0.053, 0.072)
RA SV^b^	33,272	0.017 (−0.011, 0.044)	0.030 (0.022, 0.038)
RA EF	33,272	−0.285 (−1.064, 0.495)	−0.766 (−0.991, −0.541)
LV geometric patterns			
Concentric remodeling^c^	33,237	2.12 (1.15, 3.91)	1.12 (0.87, 1.45)
Eccentric hypertrophy^c^	33,329	1.17 (0.43, 3.16)	1.59 (1.36, 1.86)
Concentric hypertrophy^c^	33,040	-	1.20 (0.68, 2.12)

a Adjusted for age, sex, body mass index, ethnicity, physical activity, Townsend deprivation index, education, smoking status, alcohol consumption status, hypertension, diabetes, high cholesterol, and metabolic syndrome.

b Log-transformed dependent variables. Exponentiation of regression coefficients is required to interpret the percent change in the outcome measure for each 1.0 unit increase in the exposure [(e^β^-1) *100].

c The effect estimates for these variables represent the hazard ratios and 95% CIs, where reference is normal LV geometry.

EDV, end-diastolic volume; EF, ejection fraction; ESV, end-systolic volume; LA, left atrium; LV, left ventricular; RA, right atrium; RV, right ventricle; and SV, stroke volume.

Similarly, as a continuous variable, FIB-4 was also associated with higher RA volumes (RA maximal volume, *β*=0.046, 95% CI 0.039–0.053; RA minimal volume, *β*=0.063, 95% CI 0.053–0.072; RA SV, *β*=0.030, 95% CI 0.022–0.038), lower LV EF (*β*=−0.276, 95% CI −0.420–−0.131), and lower RA EF (*β*=−0.766, 95% CI −0.991–−0.541). However, unlike the primary analysis, continuous FIB-4 score was not significantly associated with concentric remodeling but rather was linked to a higher risk of eccentric hypertrophy (HR = 1.59, 95% CI 1.36–1.86) (Table 3 and Supplementary Table S2).

### Exploratory cardiac pathway analysis of the FIB-4–heart disease association

Given the associations of FIB-4 with CA and HF, we combined these outcomes and explored whether adverse cardiac phenotypes statistically accounted for part of the association. Eleven CMR parameters had bootstrap confidence intervals for the indirect effect that excluded zero. The largest proportions statistically accounted for were observed for LA EF (36.38%; indirect *β*=0.0037, 95% CI 0.0026–0.0049), RA minimum volume (33.57%; indirect *β*=0.0034, 95% CI 0.0027–0.0042), and LV ESV (32.64%; indirect *β*=0.0034, 95% CI 0.0027–0.0041) (Figure 3 and Supplementary Table S3). These exploratory estimates should not be interpreted as causal mediation because temporal ordering between the CMR measures and all outcome events could not be established.

**Figure 3:**
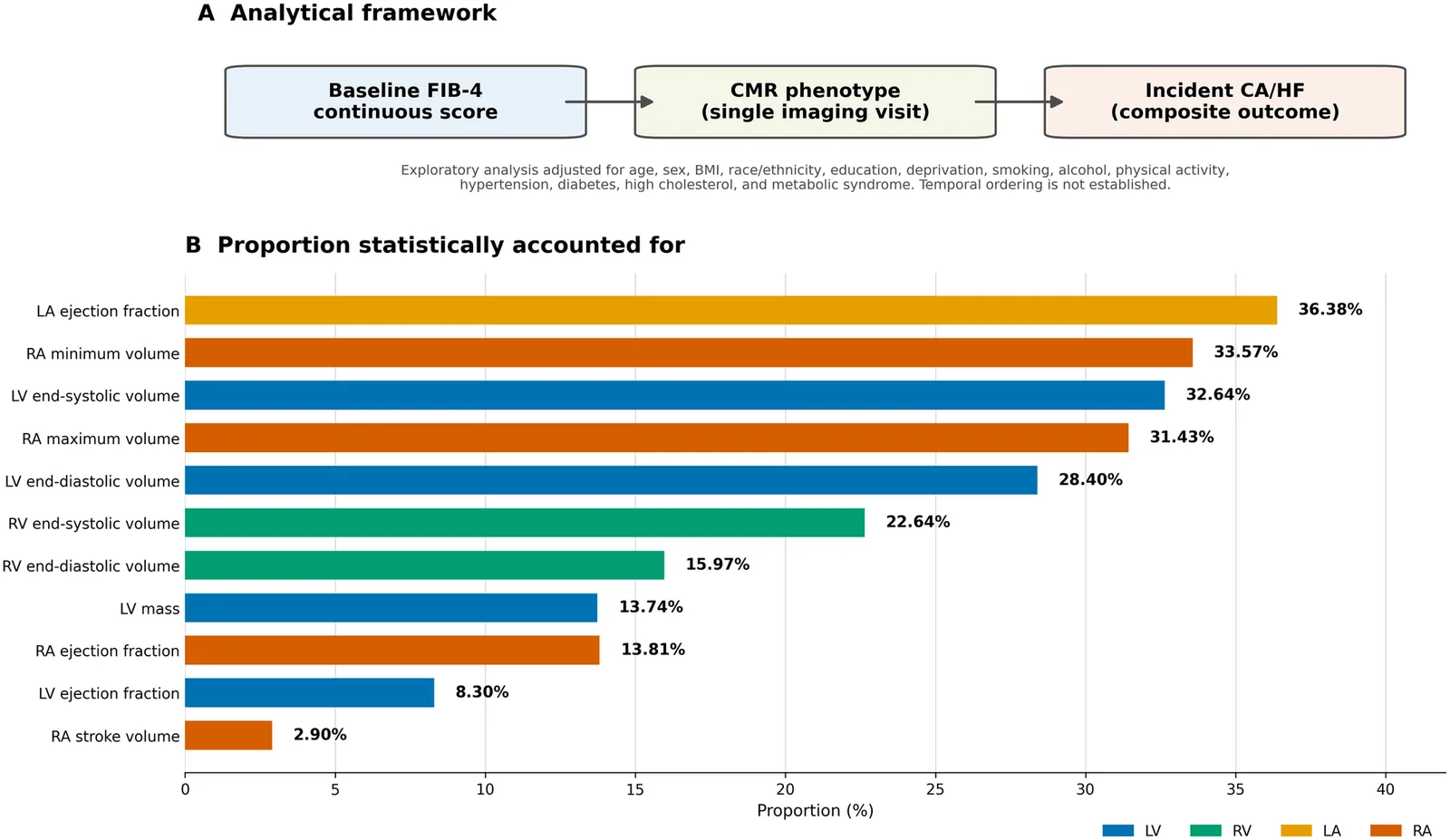
Exploratory pathway analysis of baseline FIB-4, CMR phenotypes, and incident cardiac arrhythmias/heart failure. **(A)** Analytical framework. **(B)** Proportion of the association statistically accounted for by each CMR phenotype. Models adjusted for age, sex, BMI, race/ethnicity, education, socioeconomic deprivation, smoking, alcohol consumption, physical activity, hypertension, diabetes, high cholesterol, and metabolic syndrome; 95% CIs for indirect effects were obtained from 5,000 bootstrap iterations. Temporal ordering was not established, and estimates should not be interpreted causally. CA, cardiac arrhythmias; CMR, cardiovascular magnetic resonance; EF, ejection fraction; EDV, end-diastolic volume; ESV, end-systolic volume; LA, left atrial; LV, left ventricular; RA, right atrial; RV, right ventricular.

### Supplementary analyses

Results remained consistent after excluding 40,221 participants (9.67%) with missing covariate data (Supplementary Tables S4–S5). With age-specific FIB-4 thresholds, associations persisted for overall heart disease (HR = 1.05, 95% CI 1.03–1.06), CA (HR = 1.12, 95% CI 1.09–1.15), and HF (HR = 1.07, 95% CI 1.02–1.13) in fully adjusted models (Supplementary Tables S6–S7). Similar patterns were observed using NFS (Supplementary Tables S8–S9). In age-stratified analysis, high-risk FIB-4 remained associated with CA among participants aged <65 years (HR = 1.27, 95% CI 1.17–1.38) and ≥65 years (HR = 1.18, 95% CI 1.07–1.30), with no evidence of interaction (*P* for interaction=0.29; Supplementary Table S10). Findings for HF were directionally similar (*P* for interaction=0.52). Further adjustment for statin and ACE inhibitor/ARB use produced only modest attenuation for CA (HR = 1.29, 95% CI 1.21–1.38) and HF (HR = 1.26, 95% CI 1.11–1.44), and the CMR estimates were generally similar (Supplementary Tables S11–S12).

## Discussion

### Summary of findings

In this large community-based prospective cohort of 416,001 UK Biobank participants without known heart or liver disease at baseline, higher FIB-4 was associated with incident heart disease, particularly CA and HF, and with adverse CMR phenotypes including greater LV mass, larger biventricular and biatrial volumes, lower ejection fractions, and LV geometric remodeling. Exploratory pathway analysis indicated that several CMR phenotypes statistically accounted for part of the FIB-4–CA/HF association, especially LA EF, RA minimum volume, and LV ESV (Figure 4).

**Figure 4:**
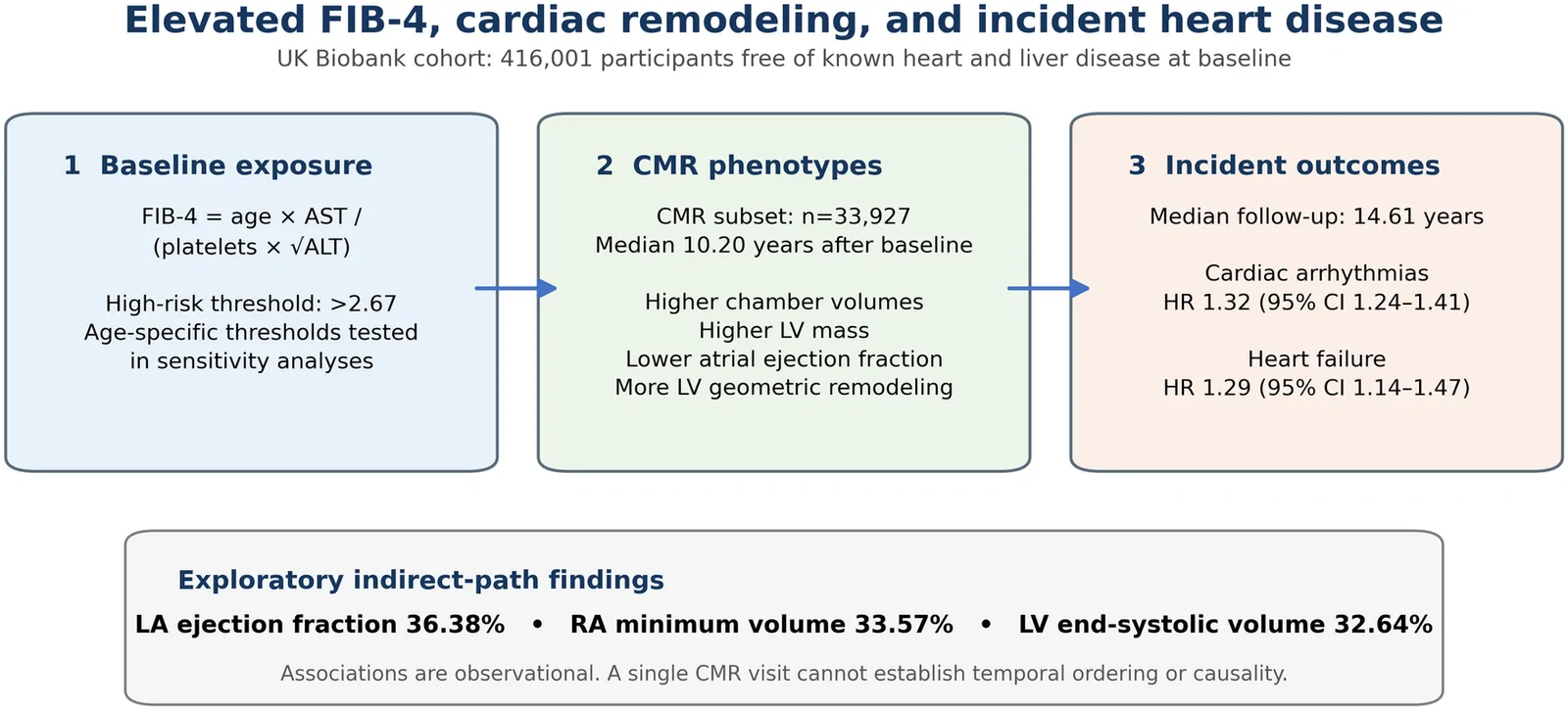
Elevated FIB-4 was associated with adverse CMR phenotypes and incident cardiac arrhythmias and heart failure. Exploratory pathway analysis identified LA ejection fraction, RA minimum volume, and LV end-systolic volume as the largest indirect statistical pathways. Associations are observational and do not establish temporal ordering or causality. CA, cardiac arrhythmias; CMR, cardiovascular magnetic resonance; EF, ejection fraction; FIB-4, Fibrosis-4 Index; HF, heart failure; LA, left atrial; LV, left ventricular; RA, right atrial.

### Association between FIB-4 and heart disease

Our findings are consistent with previous longitudinal studies linking a higher FIB-4 score to increased risk of HF, including the Veterans Aging Cohort Study (8), the Korean National Health Insurance dataset (9, 10), the Hong Kong Diabetes Register (11), and the UK Biobank (12, 15). Notably, another study from the Veterans Aging Cohort Study found association between advanced liver fibrosis and HF with preserved EF but not HF with reduced EF (13). To our knowledge, only one previous study has explored the relationship between FIB-4 and atrial fibrillation, also conducted in the UK Biobank population, but it reported no association (15). This discrepancy may be due to the study's smaller sample size, which included only approximately 30,000 participants with complete liver image-derived phenotypes for liver fat and disease activity.

### Association between FIB-4 and cardiac structure and function

Several prior observational studies have identified a link between FIB-4 and cardiac diastolic dysfunction, including E/e’ ratio, E/A ratio, deceleration time, and isovolumic relaxation time assessed by echocardiography (33–36). However, only a limited number of studies have explored the relationship between FIB-4 and CMR measures, all of which were cross-sectional and retrospective studies with small sample sizes and reported inconsistent findings. A US-based cross-sectional study (*n* = 1,668) reported an association between higher FIB-4 scores and cardiac functional abnormality, including abnormal LV EDV and RV EDV. However, no significant differences were observed for LV EF and RV EF, which aligns with our findings (37). In contrast, a retrospective cohort study from US (*n* = 66) found no association between FIB-4 score and LV EF or LV mass index, although this could be attributed to the small sample size and low statistical power in that study (38).

Our results are additionally complemented by previous echocardiogram-based studies. A cross-sectional study involving 2,161 Taiwan participants found that liver fibrosis was associated with an increase in LV mass and LA maximal volume (39). Similarly, a prospective study from the Avon Longitudinal Study of Parents and Children cohort in the south west of the UK, involving 2,047 young adults, assessed the association between transient elastography-defined liver fibrosis and cardiac measurements. Consistent with our results, this study found significant association between FIB-4 and larger LV mass index, as well as no association with LV EF. However, unlike our study, they found no correlation between liver fibrosis and LV EDV index, likely due to the limitation of 2-dimensional echocardiography in measurement of LV dimensions (40). Additionally, a cross-sectional study of 147 NAFLD patients from Italy found that severe liver fibrosis was associated with larger LA maximal volume, higher LV mass index, and lower EF. However, no significant differences were observed for LV mass. This discrepancy could be due to the limitations of echocardiogram measurements and the small sample size, which may have limited its statistical power (41).

### Exploratory cardiac pathway analysis of the FIB-4–heart disease association

Our exploratory analysis identified statistical indirect pathways through cardiac structure and function in the association of FIB-4 with CA/HF. Previous longitudinal studies have linked larger ventricular and atrial volumes, greater LV mass, and lower ejection function with future atrial fibrillation or HF (42–46). Biological mechanisms connecting hepatic injury and cardiac remodeling may include inflammation, oxidative stress, insulin resistance, and altered preload or congestion (47–49). However, the present data do not establish that elevated FIB-4 preceded the CMR changes: CMR was obtained at a single later visit, and subclinical cardiac dysfunction or venous congestion could itself influence liver enzymes and platelet count. We therefore interpret the pathway estimates as hypothesis-generating rather than causal mediation.

### Strength and limitations

Strengths include the large community-based cohort, long follow-up, linkage to clinical outcomes, and detailed CMR phenotyping. Several limitations warrant emphasis. First, the observational design precludes causal inference. Second, FIB-4 is a risk-stratification index rather than a direct measure of fibrosis. Because age and platelet count are components of FIB-4 and are independently related to cardiovascular risk, mathematical coupling and residual age- or platelet-related confounding cannot be fully excluded. We used age as the Cox-model timescale, adjusted for age in CMR and pathway models, and obtained consistent results with age-specific thresholds, which makes a purely age-driven explanation less likely but does not eliminate it. Third, the fixed >2.67 threshold favors specificity but may misclassify fibrosis risk across age groups, particularly by inflating risk classification in older adults and reducing sensitivity in younger or middle-aged adults. Fourth, CMR was measured once, a median of 10.20 years after baseline. Although baseline heart disease was excluded and FIB-4 preceded CMR, undiagnosed cardiac dysfunction or hepatic congestion could have influenced FIB-4, and the temporal order between CMR phenotypes and individual events was not fully established. Accordingly, the mediation results are exploratory. Fifth, in the medication-adjusted sensitivity models, further adjustment for baseline statin and ACE inhibitor/ARB use resulted in only modest attenuation. Nevertheless, confounding by indication and residual medication-related confounding would remain possible. SGLT2 inhibitors entered clinical use after baseline recruitment ended and therefore were not a relevant baseline covariate. Sixth, the 1.86% prevalence of high-risk FIB-4 may reflect healthy-volunteer selection in UK Biobank (50, 51). Seventh, sex-stratified analyses were not prespecified, and potential effect modification by sex requires further investigation. Eighth, given the large sample and multiple cardiac phenotypes examined, some statistically significant associations of small magnitude may have limited clinical relevance. Because no multiplicity adjustment was applied, secondary CMR and pathway findings should be interpreted cautiously. Finally, residual and unmeasured confounding cannot be excluded.

## Conclusion

In this cohort study, higher FIB-4 was associated with incident CA and HF and with adverse CMR markers of cardiac structure and function. Cardiac chamber enlargement and impaired atrial function were statistically consistent with indirect pathways linking FIB-4 to CA/HF, but the data do not establish temporal ordering or causality. FIB-4 may complement cardiovascular risk assessment, although confirmation with direct liver-fibrosis measures and longitudinal cardiac imaging is needed before clinical implementation.

## Data Availability

Publicly available datasets were analyzed in this study. This data can be found here: the UK Biobank repository, http://www.ukbiobank.ac.uk.
