# Supplementary material for: Linking liver fibrosis to cardiac structure, function, and heart disease: a community-based fibrosis-4 index study
Source: Front Cardiovasc Med. 2026 Oct 7;13:1777872. doi: 10.3389/fcvm.2026.1777872 (PMC13683155; doi:10.3389/fcvm.2026.1777872)
Supplement: Supplementary file 1 [file Datasheet1.docx]

**Supplemental materials**

**Table S1.** Basic-adjusted hazard ratios (HRs), 20th percentile differences (PDs, years), and 95% confidence intervals (95% CIs) for association between the Fibrosis-4 Index (FIB-4) and different forms of heart disease.

**Table S2.** Basic-adjusted *β* Coefficients and 95% Confidence Intervals (CIs) for the association between the Fibrosis-4 Index (FIB-4) and cardiac phenotypes.

**Table S3.** Exploratory indirect effects through cardiac structure and function in the association between the Fibrosis-4 Index (FIB-4) and cardiac arrhythmias or heart failure.

**Table S4.** Hazard ratios (HRs) and 95% confidence intervals (95% CIs) for the association between the Fibrosis-4 Index (FIB-4) and different forms of heart disease including only participants with complete data.

**Table S5.** *β* Coefficients and 95% confidence intervals (95% CIs) for the association between the Fibrosis-4 Index (FIB-4) and cardiac phenotypes including only participants with complete data.

**Table S6.** Hazard ratios (HRs) and 95% confidence intervals (95% CIs) for associations of age-specific FIB-4 risk categories with incident heart disease outcomes.

**Table S7.** *β* coefficients and 95% confidence intervals (95% CIs) for associations of age-specific FIB-4 risk categories with CMR phenotypes.

**Table S8.** Hazard ratios (HRs) and 95% confidence intervals (95% CIs) for the association between the Fibrosis-4 Index (FIB-4) and different subtypes of heart disease using the Nonalcoholic Fatty Liver Disease Fibrosis Score.

**Table S9.** *β* Coefficients and 95% confidence intervals (95% CIs) for the association between the Fibrosis-4 Index (FIB-4) and cardiac phenotypes using the Nonalcoholic Fatty Liver Disease Fibrosis Score.

**Table S10.** Age-stratified associations of high-risk FIB-4 (>2.67) with incident heart disease.

**Table S11.** Medication-adjusted associations of FIB-4 with incident heart disease.

**Table S12.** Medication-adjusted associations of FIB-4 with CMR phenotypes.

**Table S1.** Basic-adjusted hazard ratios (HRs), 20th percentile differences (PDs, years), and 95% confidence intervals (95% CIs) for the association between the Fibrosis-4 Index (FIB-4) and different forms of heart disease.

| **Heart disease** | **No. of case** | **Primary approach: High-risk versus low/indeterminate-risk FIB-4** | | **Secondary approach: For each 1.0 unit increase in FIB-4** | |
| --- | --- | --- | --- | --- | --- |
|  |  | **Basic-adjusted**  **HR (95% CI)^*^** | **Basic-adjusted**  **20th PDs (95% CI)^*^** | **Basic-adjusted**  **HR (95% CI)^*^** | **Basic-adjusted**  **20th PDs (95% CI)^*^** |
| **All-cause heart disease** | 57,207 | 1.19 (1.13, 1.24) | -1.85 (-2.29, -1.41) | 1.05 (1.04, 1.07) | -0.57 (-0.70, -0.44) |
| **Coronary heart disease** | 29,785 | 1.03 (0.96, 1.10) | -0.48 (-1.10, 0.13) | 1.06 (1.04, 1.08) | -0.61 (-0.78, -0.44) |
| Angina pectoris | 9,246 | 0.97 (0.85, 1.11) | 0.09 (-0.96, 1.15) | 1.02 (0.97, 1.06) | -0.28 (-0.61, 0.05) |
| Myocardial infarction | 8,523 | 1.04 (0.90, 1.20) | -0.44 (-1.43, 0.54) | 0.96 (0.92, 1.003) | 0.12 (-0.20, 0.45) |
| Other acute ischaemic heart diseases | 1,290 | 1.14 (0.81, 1.59) | -0.91 (-2.75, 0.94) | 1.09 (0.98, 1.20) | -0.62 (-1.24, -0.004) |
| Chronic ischaemic heart disease | 19,776 | 1.03 (0.94, 1.12) | -0.35 (-0.99, 0.30) | 1.09 (1.06, 1.12) | -0.73 (-0.92, -0.54) |
| **Cardiac arrhythmias** | 25,651 | 1.33 (1.24, 1.41) | -2.30 (-2.88, -1.73) | 1.16 (1.14, 1.19) | -1.31 (-1.50, -1.12) |
| Atrial fibrillation and flutter | 21,360 | 1.34 (1.25, 1.44) | -2.32 (-2.86, -1.79) | 1.17 (1.15, 1.20) | -1.31 (-1.49, -1.12) |
| Other cardiac arrhythmias | 4,663 | 1.26 (1.06, 1.50) | -1.71 (-2.85, -0.58) | 1.11 (1.06, 1.18) | -0.88 (-1.20, -0.56) |
| **Heart failure** | 6,586 | 1.32 (1.16, 1.50) | -1.76 (-2.55, -0.98) | 1.10 (1.06, 1.15) | -0.69 (-0.97, -0.41) |

^*^Adjusted for age and sex.

**Table S2.** Basic-adjusted *β* Coefficients and 95% Confidence Intervals (CIs) for the association between the Fibrosis-4 Index (FIB-4) and cardiac phenotypes.

| **Phenotype** | **No. of subject** | **Primary approach: High-risk versus low/indeterminate-risk FIB-4** | **Secondary approach: For each 1.0 unit increase in FIB-4** |
| --- | --- | --- | --- |
|  |  | **Basic-adjusted *β* (95% CI)^*^** | **Basic-adjusted *β* (95% CI)^*^** |
| LV EDV^†^ | 33,624 | 0.032 (0.017, 0.047) | 0.037 (0.033, 0.041) |
| LV ESV^†^ | 33,624 | 0.043 (0.023, 0.064) | 0.044 (0.039, 0.050) |
| LV SV^†^ | 33,624 | 0.023 (0.007, 0.039) | 0.032 (0.027, 0.036) |
| LV mass^†^ | 33,626 | 0.010 (-0.003, 0.024) | 0.017 (0.013, 0.021) |
| LV EF | 33,624 | -0.493 (-0.996, 0.009) | -0.308 (-0.452, -0.163) |
| RV EDV^†^ | 33,624 | 0.024 (0.009, 0.039) | 0.038 (0.033, 0.042) |
| RV ESV^†^ | 33,624 | 0.023 (0.003, 0.043) | 0.042 (0.037, 0.048) |
| RV SV^†^ | 33,624 | 0.025 (0.007, 0.042) | 0.035 (0.030, 0.040) |
| RV EF | 33,624 | 0.049 (-0.453, 0.551) | -0.190 (-0.335, -0.046) |
| LA maximal volume^†^ | 33,272 | 0.051 (0.025, 0.076) | 0.048 (0.040, 0.055) |
| LA minimal volume^†^ | 33,272 | 0.084 (0.040, 0.129) | 0.075 (0.062, 0.087) |
| LA SV^†^ | 33,272 | 0.037 (0.015, 0.060) | 0.034 (0.027, 0.040) |
| LA EF | 33,272 | -0.934 (-1.725, -0.143) | -0.858 (-1.086, -0.630) |
| RA maximal volume^†^ | 33,272 | 0.022 (-0.003, 0.046) | 0.054 (0.047, 0.061) |
| RA minimal volume^†^ | 33,272 | 0.032 (-0.001, 0.065) | 0.072 (0.062, 0.082) |
| RA SV^†^ | 33,272 | 0.016 (-0.012, 0.044) | 0.036 (0.028, 0.044) |
| RA EF | 33,272 | -0.330 (-1.112, 0.451) | -0.843 (-1.069, -0.618) |
| LV geometric patterns |  |  |  |
| Concentric remodeling^‡^ | 33,237 | 2.18 (1.18, 4.02) | 1.02 (0.78, 1.34) |
| Eccentric hypertrophy^‡^ | 33,329 | 1.08 (0.40, 2.92) | 1.53 (1.32, 1.78) |
| Concentric hypertrophy^‡^ | 33,040 | - | 1.18 (0.66, 2.09) |

^*^Adjusted for age and sex.

^†^Log-transformed dependent variables. Exponentiation of regression coefficients is required to interpret the percent change in the outcome measure for each 1.0 unit increase in the exposure [(e^β^-1)^*^100].

^‡^The effect estimates for these variables represent the hazard ratios and 95% CIs, where reference is normal LV geometry.

Abbreviations: EDV, end-diastolic volume; EF, ejection fraction; ESV, end-systolic volume; LA, left atrium; LV, left ventricle; RA, right atrium; RV, right ventricle; and SV, stroke volume.

**Table S3. Exploratory indirect effects through cardiac structure and function in the association between the Fibrosis-4 Index (FIB-4) and cardiac arrhythmias or heart failure.**

| **Potential indirect pathways** | **Cardiac arrhythmias or heart failure** | |
| --- | --- | --- |
|  | **Fully adjusted *β*-coefficient (95% CI)^*^** | ***P* value** |
| **LV EDV** |  |  |
| Direct effects | 0.0074 (0.0009, 0.0139) | 0.027 |
| Indirect effects | 0.0029 (0.0023, 0.0036) | <0.001 |
| Total effects | 0.0103 (0.0038, 0.0168) | 0.002 |
| **LV ESV** |  |  |
| Direct effects | 0.0070 (0.0005, 0.0134) | 0.036 |
| Indirect effects | 0.0034 (0.0027, 0.0041) | <0.001 |
| Total effects | 0.0103 (0.0038, 0.0168) | 0.002 |
| **LV SV** |  |  |
| Direct effects | 0.0100 (0.0035, 0.0165) | 0.003 |
| Indirect effects | 0.0003 (-0.0001, 0.0007) | 0.173 |
| Total effects | 0.0103 (0.0038, 0.0168) | 0.002 |
| **LV mass** |  |  |
| Direct effects | 0.0089 (0.0023, 0.0155) | 0.008 |
| Indirect effects | 0.0014 (0.0010, 0.0019) | <0.001 |
| Total effects | 0.0103 (0.0038, 0.0169) | 0.002 |
| **LV EF** |  |  |
| Direct effects | 0.0095 (0.0030, 0.0159) | 0.004 |
| Indirect effects | 0.0009 (0.0004, 0.0014) | 0.001 |
| Total effects | 0.0103 (0.0038, 0.0168) | 0.002 |
| **RV EDV** |  |  |
| Direct effects | 0.0087 (0.0022, 0.0152) | 0.009 |
| Indirect effects | 0.0016 (0.0011, 0.0022) | <0.001 |
| Total effects | 0.0103 (0.0038, 0.0168) | 0.002 |
| **RV ESV** |  |  |
| Direct effects | 0.0080 (0.0015, 0.0144) | 0.016 |
| Indirect effects | 0.0023 (0.0018, 0.0030) | <0.001 |
| Total effects | 0.0103 (0.0038, 0.0168) | 0.002 |
| **RV SV** |  |  |
| Direct effects | 0.0101 (0.0035, 0.0166) | <0.001 |
| Indirect effects | 0.0003 (-0.0006, 0.0011) | 0.201 |
| Total effects | 0.0103 (0.0038, 0.0168) | 0.002 |
| **RV EF** |  |  |
| Direct effects | 0.0099 (0.0034, 0.0163) | 0.003 |
| Indirect effects | 0.0004 (0.00002, 0.0008) | 0.081 |
| Total effects | 0.0103 (0.0038, 0.0168) | 0.002 |
| **LA maximal volume** |  |  |
| Direct effects | 0.0060 (-0.0003, 0.0128) | 0.073 |
| Indirect effects | 0.0041 (0.0032, 0.0051) | <0.001 |
| Total effects | 0.0101 (0.0039, 0.0168) | 0.002 |
| **LA minimal volume** |  |  |
| Direct effects | 0.0058 (-0.0005, 0.0124) | 0.080 |
| Indirect effects | 0.0043 (0.0034, 0.0053) | <0.001 |
| Total effects | 0.01010 (0.0039, 0.0168) | 0.002 |
| **LA SV** |  |  |
| Direct effects | 0.0101 (0.0038, 0.0168) | 0.002 |
| Indirect effects | 0.00003 (-0.0003, 0.0003) | 0.833 |
| Total effects | 0.0101 (0.0039, 0.0168) | 0.002 |
| **LA EF** |  |  |
| Direct effects | 0.0064 (0.0003, 0.0131) | 0.049 |
| Indirect effects | 0.0037 (0.0026, 0.0049) | <0.001 |
| Total effects | 0.0101 (0.0039, 0.0168) | 0.002 |
| **RA maximal volume** |  |  |
| Direct effects | 0.0069 (0.0006, 0.0138) | 0.038 |
| Indirect effects | 0.0032 (0.0025, 0.0039) | <0.001 |
| Total effects | 0.0101 (0.0039, 0.0168) | 0.002 |
| **RA minimal volume** |  |  |
| Direct effects | 0.0067 (0.0004, 0.0135) | 0.044 |
| Indirect effects | 0.0034 (0.0027, 0.0042) | <0.001 |
| Total effects | 0.0101 (0.0039, 0.0168) | 0.002 |
| **RA SV** |  |  |
| Direct effects | 0.0098 (0.0036, 0.0165) | 0.003 |
| Indirect effects | 0.0003 (0.0001, 0.0006) | 0.021 |
| Total effects | 0.0101 (0.0039, 0.0168) | 0.002 |
| **RA EF** |  |  |
| Direct effects | 0.0087 (0.0024, 0.0154) | 0.009 |
| Indirect effects | 0.0014 (0.0010, 0.0019) | <0.001 |
| Total effects | 0.0101 (0.0039, 0.0168) | 0.002 |

^*^Adjusted for age, sex, body mass index, ethnicity, education, Townsend deprivation index, smoking status, alcohol consumption status, physical activity, diabetes, high cholesterol, and metabolic syndrome.

Abbreviations: LV, left ventricular; EDV, end-diastolic volume; EF, ejection fraction; ESV, end-systolic volume; LA, left atrium; LV, left ventricular; RA, right atrium; RV, right ventricle; and SV, stroke volume.

**Table S4.** Hazard ratios (HRs) and 95% confidence intervals (95% CIs) for the association between the Fibrosis-4 Index (FIB-4) and different forms of heart disease including only participants with complete data.

| **Heart disease** | **No. of case** | **Primary approach: High-risk versus low/indeterminate-risk FIB-4** | | **Secondary approach: For each 1.0 unit increase in FIB-4** | |
| --- | --- | --- | --- | --- | --- |
|  |  | **Basic-adjusted**  **HR (95% CI)^*^** | **Multi-adjusted**  **HR (95% CI)**^†^ | **Basic-adjusted**  **HR (95% CI)^*^** | **Multi-adjusted**  **HR (95% CI)**^†^ |
| **All-cause heart disease** | 50,149 | 1.18 (1.13, 1.25) | 1.18 (1.12, 1.24) | 1.06 (1.04, 1.08) | 1.08 (1.06, 1.09) |
| **Coronary heart disease** | 26,004 | 1.02 (0.95, 1.10) | 1.02 (0.94, 1.10) | 1.06 (1.04, 1.09) | 1.08 (1.06, 1.11) |
| Angina pectoris | 8,091 | 1.01 (0.88, 1.16) | 1.01 (0.87, 1.16) | 0.94 (0.89, 0.98) | 0.97 (0.92, 1.01) |
| Myocardial infarction | 7,495 | 1.05 (0.90, 1.23) | 1.02 (0.88, 1.19) | 0.97 (0.92, 1.01) | 1.00 (0.95, 1.05) |
| Other acute ischaemic heart diseases | 1,134 | 0.99 (0.68, 1.46) | 0.98 (0.67, 1.44) | 1.03 (0.92, 1.16) | 1.06 (0.95, 1.19) |
| Chronic ischaemic heart disease | 17,312 | 1.003 (0.91, 1.10) | 0.99 (0.91, 1.09) | 1.09 (1.06, 1.12) | 1.10 (1.07, 1.13) |
| **Cardiac arrhythmias** | 22,794 | 1.34 (1.25, 1.44) | 1.33 (1.24, 1.43) | 1.17 (1.15, 1.20) | 1.18 (1.15, 1.20) |
| Atrial fibrillation and flutter | 18,908 | 1.36 (1.26, 1.46) | 1.35 (1.25, 1.45) | 1.18 (1.15, 1.21) | 1.18 (1.16, 1.21) |
| Other cardiac arrhythmias | 4,219 | 1.28 (1.07, 1.53) | 1.27 (1.06, 1.52) | 1.13 (1.07, 1.20) | 1.14 (1.08, 1.20) |
| **Heart failure** | 5,541 | 1.30 (1.13, 1.50) | 1.27 (1.11, 1.47) | 1.10 (1.05, 1.16) | 1.13 (1.08, 1.18) |

^*^Adjusted for age and sex.

^†^Adjusted for age, sex, body mass index, ethnicity, physical activity, Townsend deprivation index, education, smoking status, alcohol consumption status, hypertension, diabetes, high cholesterol, and metabolic syndrome.

**Table S5.** *β* Coefficients and 95% confidence intervals (95% CIs) for the association between the Fibrosis-4 Index (FIB-4) and cardiac phenotypes including only participants with complete data.

|  |  | **Primary approach: High-risk versus low/indeterminate-risk FIB-4** | | **Secondary approach: For each 1.0 unit increase in FIB-4** | |
| --- | --- | --- | --- | --- | --- |
| **Phenotype** | **No. of subject** | **Basic-adjusted**  ***β* (95% CI)^*^** | **Multi-adjusted**  ***β* (95% CI)**^†^ | **Basic-adjusted**  ***β* (95% CI)^*^** | **Multi-adjusted**  ***β* (95% CI)**^†^ |
| LV EDV^‡^ | 32,182 | 0.034 (0.019, 0.050) | 0.035 (0.020, 0.050) | 0.038 (0.033, 0.042) | 0.034 (0.029, 0.038) |
| LV ESV^‡^ | 32,182 | 0.047 (0.026, 0.068) | 0.048 (0.027, 0.068) | 0.045 (0.039, 0.052) | 0.041 (0.035, 0.047) |
| LV SV^‡^ | 32,182 | 0.025 (0.008, 0.042) | 0.026 (0.010, 0.043) | 0.032 (0.027, 0.037) | 0.029 (0.024, 0.034) |
| LV mass^‡^ | 32,184 | 0.014 (0.0003, 0.027) | 0.015 (0.001, 0.028) | 0.017 (0.013, 0.021) | 0.017 (0.013, 0.021) |
| LV EF | 32,182 | -0.523 (-1.036, -0.010) | -0.505 (-1.018, 0.007) | -0.317 (-0.465, -0.168) | -0.283 (-0.431, -0.134) |
| RV EDV^‡^ | 32,182 | 0.025 (0.010, 0.041) | 0.026 (0.011, 0.041) | 0.038 (0.034, 0.043) | 0.034 (0.029, 0.038) |
| RV ESV^‡^ | 32,182 | 0.024 (0.004, 0.045) | 0.024 (0.004, 0.045) | 0.044 (0.038, 0.050) | 0.037 (0.031, 0.043) |
| RV SV^‡^ | 32,182 | 0.026 (0.009, 0.044) | 0.028 (0.010, 0.046) | 0.035 (0.030, 0.040) | 0.031 (0.026, 0.036) |
| RV EF | 32,182 | 0.052 (-0.461, 0.564) | 0.091 (-0.419, 0.601) | -0.217 (-0.365, -0.069) | -0.153 (-0.301, -0.006) |
| LA maximal volume^‡^ | 31,840 | 0.057 (0.031, 0.084) | 0.058 (0.031, 0.084) | 0.049 (0.041, 0.056) | 0.044 (0.037, 0.052) |
| LA minimal volume^‡^ | 31,840 | 0.096 (0.050, 0.141) | 0.095 (0.050, 0.140) | 0.075 (0.062, 0.089) | 0.070 (0.057, 0.083) |
| LA SV^‡^ | 31,840 | 0.041 (0.017, 0.064) | 0.041 (0.018, 0.064) | 0.034 (0.027, 0.041) | 0.030 (0.024, 0.037) |
| LA EF | 31,840 | -1.117 (-1.923, -0.311) | -1.085 (-1.891, -0.279) | -0.879 (-1.112, -0.646) | -0.845 (-1.079, -0.610) |
| RA maximal volume^†^ | 31,840 | 0.023 (-0.002, 0.049) | 0.023 (-0.002, 0.048) | 0.056 (0.049, 0.063) | 0.048 (0.041, 0.055) |
| RA minimal volume^†^ | 31,840 | 0.032 (-0.002, 0.067) | 0.031 (-0.002, 0.065) | 0.074 (0.064, 0.084) | 0.064 (0.055, 0.074) |
| RA SV^‡^ | 31,840 | 0.018 (-0.010, 0.047) | 0.019 (-0.009, 0.047) | 0.038 (0.030, 0.046) | 0.032 (0.024, 0.040) |
| RA EF | 31,840 | -0.290 (-1.090, 0.509) | -0.247 (-1.044, 0.551) | -0.833 (-1.064, -0.601) | -0.749 (-0.981, -0.517) |
| LV geometric patterns |  |  |  |  |  |
| Concentric remodeling^§^ | 31,809 | 2.06 (1.08, 3.92) | 1.97 (1.03, 3.74) | 0.98 (0.74, 1.30) | 1.08 (0.83, 1.41) |
| Eccentric hypertrophy^§^ | 31,903 | 1.13 (0.42, 3.06) | 1.21 (0.45, 3.27) | 1.65 (1.38, 1.97) | 1.66 (1.39, 1.98) |
| Concentric hypertrophy^§^ | 31,624 | - | - | 1.08 (0.58, 2.02) | 1.12 (0.61, 2.06) |

^*^Adjusted for age and sex.

^†^Adjusted for age, sex, body mass index, ethnicity, physical activity, Townsend deprivation index, education, smoking status, alcohol consumption status, hypertension, diabetes, high cholesterol, and metabolic syndrome.

^‡^Log-transformed dependent variables. Exponentiation of regression coefficients is required to interpret the percent change in the outcome measure for each 1.0 unit increase in the exposure [(e^β^-1)^*^100].

^§^The effect estimates for these variables represent the hazard ratios and 95% CIs, where reference is normal LV geometry.

Abbreviations: EDV, end-diastolic volume; EF, ejection fraction; ESV, end-systolic volume; LA, left atrium; LV, left ventricle; RA, right atrium; RV, right ventricle; and SV, stroke volume.

**Table S6. Hazard ratios (HRs) and 95% confidence intervals (95% CIs) for associations of age-specific FIB-4 risk categories with incident heart disease outcomes.**

| **Heart disease** | **No. of case** | **Primary approach: High-risk versus lower-risk age-specific FIB-4 categories** | |
| --- | --- | --- | --- |
|  |  | **Basic-adjusted**  **HR (95% CI)^*^** | **Multi-adjusted**  **HR (95% CI)^†^** |
| **All-cause heart disease** | 57,207 | 1.02 (1.003, 1.04) | 1.05 (1.03, 1.06) |
| **Coronary heart disease** | 29,785 | 1.03 (1.01, 1.06) | 1.06 (1.04, 1.09) |
| Angina pectoris | 9,246 | 1.001 (0.96, 1.05) | 1.03 (0.98, 1.07) |
| Myocardial infarction | 8,523 | 0.96 (0.92, 1.003) | 0.99 (0.95, 1.04) |
| Other acute ischaemic heart diseases | 1,290 | 1.01 (0.90, 1.13) | 1.05 (0.93, 1.17) |
| Chronic ischaemic heart disease | 19,776 | 1.06 (1.03, 1.09) | 1.09 (1.06, 1.12) |
| **Cardiac arrhythmias** | 25,651 | 1.11 (1.08, 1.14) | 1.12 (1.09, 1.15) |
| Atrial fibrillation and flutter | 21,360 | 1.13 (1.10, 1.16) | 1.14 (1.11, 1.17) |
| Other cardiac arrhythmias | 4,663 | 1.06 (1.002, 1.13) | 1.07 (1.01, 1.14) |
| **Heart failure** | 6,586 | 1.03 (0.98, 1.09) | 1.07 (1.02, 1.13) |

**^*^**Adjusted for age and sex.

**^†^**Adjusted for age, sex, body mass index, ethnicity, physical activity, Townsend deprivation index, education, smoking status, alcohol consumption status, hypertension, diabetes, high cholesterol, and metabolic syndrome.

**Table S7. β coefficients and 95% confidence intervals (95% CIs) for associations of age-specific FIB-4 risk categories with CMR phenotypes.**

| **Phenotype** | **No. of subject** | **Primary approach: High-risk versus lower-risk age-specific FIB-4 categories** | |
| --- | --- | --- | --- |
|  |  | **Basic-adjusted**  ***β* (95% CI)^*^** | **Multi-adjusted**  ***β* (95% CI)**^†^ |
| LV EDV^‡^ | 33,624 | 0.030 (0.026, 0.034) | 0.027 (0.023, 0.031) |
| LV ESV^‡^ | 33,624 | 0.032 (0.026, 0.037) | 0.027 (0.022, 0.033) |
| LV SV^‡^ | 33,624 | 0.029 (0.024, 0.033) | 0.026 (0.021, 0.030) |
| LV mass^‡^ | 33,626 | 0.015 (0.011, 0.018) | 0.014 (0.011, 0.018) |
| LV EF | 33,624 | -0.072 (-0.207, 0.064) | -0.044 (-0.180, 0.092) |
| RV EDV^‡^ | 33,624 | 0.032 (0.028, 0.036) | 0.028 (0.024, 0.032) |
| RV ESV^‡^ | 33,624 | 0.034 (0.028, 0.039) | 0.028 (0.023, 0.034) |
| RV SV^‡^ | 33,624 | 0.032 (0.027, 0.037) | 0.029 (0.024, 0.033) |
| RV EF | 33,624 | -0.043 (-0.178, 0.093) | 0.005 (-0.130, 0.140) |
| LA maximal volume^‡^ | 33,272 | 0.034 (0.027, 0.041) | 0.031 (0.024, 0.038) |
| LA minimal volume^‡^ | 33,272 | 0.050 (0.038, 0.062) | 0.046 (0.034, 0.058) |
| LA SV^‡^ | 33,272 | 0.026 (0.020, 0.032) | 0.022 (0.016, 0.029) |
| LA EF | 33,272 | -0.537 (-0.751, -0.323) | -0.517 (-0.732, -0.303) |
| RA maximal volume^†^ | 33,272 | 0.046 (0.039, 0.053) | 0.039 (0.033, 0.046) |
| RA minimal volume^†^ | 33,272 | 0.062 (0.053, 0.071) | 0.054 (0.045, 0.063) |
| RA SV^‡^ | 33,272 | 0.030 (0.023, 0.038) | 0.025 (0.017, 0.032) |
| RA EF | 33,272 | -0.750 (-0.962, -0.538) | -0.688 (-0.899, -0.476) |
| LV geometric patterns |  |  |  |
| Concentric remodeling^§^ | 33,237 | 0.90 (0.69, 1.17) | 0.97 (0.74, 1.27) |
| Eccentric hypertrophy^§^ | 33,329 | 1.81 (1.43, 2.28) | 1.85 (1.46, 2.33) |
| Concentric hypertrophy^§^ | 33,040 | 1.29 (0.72, 2.32) | 1.32 (0.73, 2.38) |

^*^Adjusted for age and sex.

^†^Adjusted for age, sex, body mass index, ethnicity, physical activity, Townsend deprivation index, education, smoking status, alcohol consumption status, hypertension, diabetes, high cholesterol, and metabolic syndrome.

^‡^Log-transformed dependent variables. Exponentiation of regression coefficients is required to interpret the percent change in the outcome measure for each 1.0 unit increase in the exposure [(e^β^-1)^*^100].

^§^The effect estimates for these variables represent the hazard ratios and 95% CIs, where reference is normal LV geometry.

Abbreviations: EDV, end-diastolic volume; EF, ejection fraction; ESV, end-systolic volume; LA, left atrium; LV, left ventricle; RA, right atrium; RV, right ventricle; and SV, stroke volume.

**Table S8.** Hazard ratios (HRs) and 95% confidence intervals (95% CIs) for the association between the Fibrosis-4 Index (FIB-4) and different subtypes of heart disease using the Nonalcoholic Fatty Liver Disease Fibrosis Score.

| **Heart disease** | **No. of case** | **Primary approach: High-risk versus lower-risk NFS categories** | |
| --- | --- | --- | --- |
|  |  | **Basic-adjusted**  **HR (95% CI)^*^** | **Multi-adjusted**  **HR (95% CI)^†^** |
| **All-cause heart disease** | 52,555 | 1.16 (1.11, 1.21) | 1.13 (1.08, 1.18) |
| **Coronary heart disease** | 27,392 | 1.05 (0.98, 1.12) | 1.01 (0.94, 1.07) |
| Angina pectoris | 8,485 | 0.92 (0.82, 1.04) | 0.90 (0.80, 1.02) |
| Myocardial infarction | 7,832 | 0.91 (0.80, 1.04) | 0.90 (0.78, 1.02) |
| Other acute ischaemic heart diseases | 1,177 | 1.34 (0.996, 1.80) | 1.26 (0.94, 1.70) |
| Chronic ischaemic heart disease | 18,206 | 1.08 (0.999, 1.16) | 1.02 (0.95, 1.11) |
| **Cardiac arrhythmias** | 23,522 | 1.27 (1.20, 1.35) | 1.27 (1.19, 1.35) |
| Atrial fibrillation and flutter | 19,595 | 1.26 (1.18, 1.34) | 1.25 (1.17, 1.33) |
| Other cardiac arrhythmias | 4,262 | 1.21 (1.01, 1.44) | 1.22 (1.02, 1.45) |
| **Heart failure** | 6,050 | 1.39 (1.26, 1.55) | 1.28 (1.15, 1.42) |

**^*^**Adjusted for age and sex.

**^†^**Adjusted for age, body mass index, sex, ethnicity, physical activity, Townsend deprivation index, education, smoking status, alcohol consumption status, hypertension, diabetes, high cholesterol, and metabolic syndrome.

**Table S9.** *β* Coefficients and 95% confidence intervals (95% CIs) for the association between the Fibrosis-4 Index (FIB-4) and cardiac phenotypes using the Nonalcoholic Fatty Liver Disease Fibrosis Score.

| **Phenotype** | **No. of subject** | **Primary approach: High-risk versus lower-risk NFS categories** | |
| --- | --- | --- | --- |
|  |  | **Basic-adjusted**  ***β* (95% CI)^*^** | **Multi-adjusted**  ***β* (95% CI)**^†^ |
| LV EDV^‡^ | 30,706 | 0.018 (0.001, 0.036) | 0.025 (0.007, 0.042) |
| LV ESV^‡^ | 30,706 | 0.034 (0.009, 0.058) | 0.038 (0.013, 0.062) |
| LV SV^‡^ | 30,706 | 0.007 (-0.012, 0.027) | 0.016 (-0.003, 0.035) |
| LV mass^‡^ | 30,708 | -0.014 (-0.030, 0.002) | -0.006 (-0.022, 0.009) |
| LV EF | 30,706 | -0.629 (-1.226, -0.033) | -0.536 (-1.133, 0.061) |
| RV EDV^‡^ | 30,706 | 0.017 (-0.001, 0.035) | 0.023 (0.006, 0.041) |
| RV ESV^‡^ | 30,706 | 0.030 (0.006, 0.054) | 0.032 (0.008, 0.055) |
| RV SV^‡^ | 30,706 | 0.009 (-0.012, 0.029) | 0.017 (-0.003, 0.038) |
| RV EF | 30,706 | -0.528 (-1.123, 0.066) | -0.365 (-0.958, 0.228) |
| LA maximal volume^‡^ | 30,370 | 0.024 (-0.007, 0.055) | 0.032 (0.001, 0.063) |
| LA minimal volume^‡^ | 30,370 | 0.063 (0.010, 0.116) | 0.069 (0.016, 0.122) |
| LA SV^‡^ | 30,370 | -0.003 (-0.030, 0.024) | 0.006 (-0.021, 0.032) |
| LA EF | 30,370 | -1.493 (-2.435, -0.551) | -1.419 (-2.363, -0.476) |
| RA maximal volume^†^ | 30,370 | 0.045 (0.016, 0.074) | 0.050 (0.021, 0.079) |
| RA minimal volume^†^ | 30,370 | 0.070 (0.030, 0.109) | 0.074 (0.035, 0.113) |
| RA SV^‡^ | 30,370 | 0.022 (-0.012, 0.055) | 0.028 (-0.006, 0.061) |
| RA EF | 30,370 | -1.048 (-1.977, -0.118) | -0.996 (-1.926, -0.067) |
| LV geometric patterns |  |  |  |
| Concentric remodeling^§^ | 30,359 | 0.90 (0.40, 2.02) | 0.96 (0.43, 2.14) |
| Eccentric hypertrophy^§^ | 30,428 | 0.68 (0.17, 2.81) | 0.80 (0.19, 3.28) |
| Concentric hypertrophy^§^ | 30,171 | 0.82 (0.16, 4.11) | 0.98 (0.19, 5.06) |

^*^Adjusted for age and sex.

^†^Adjusted for age, sex, body mass index, ethnicity, physical activity, Townsend deprivation index, education, smoking status, alcohol consumption status, hypertension, diabetes, high cholesterol, and metabolic syndrome.

^‡^Log-transformed dependent variables. Exponentiation of regression coefficients is required to interpret the percent change in the outcome measure for each 1.0 unit increase in the exposure [(e^β^-1)^*^100].

^§^The effect estimates for these variables represent the hazard ratios and 95% CIs, where reference is normal LV geometry.

Abbreviations: EDV, end-diastolic volume; EF, ejection fraction; ESV, end-systolic volume; LA, left atrium; LV, left ventricle; RA, right atrium; RV, right ventricle; and SV, stroke volume.

**Table S10 Age-stratified associations of high-risk FIB-4 (>2.67) with incident heart disease.**

| **Outcome** | **Age stratum** | **Fully adjusted HR (95% CI)** | ***P* for interaction** |
| --- | --- | --- | --- |
| All heart disease | <65 years | 1.15 (1.08–1.22) | 0.18 |
| All heart disease | ≥65 years | 1.08 (1.01–1.15) |  |
| Cardiac arrhythmias | <65 years | 1.27 (1.17–1.38) | 0.29 |
| Cardiac arrhythmias | ≥65 years | 1.18 (1.07–1.30) |  |
| Heart failure | <65 years | 1.24 (1.04–1.48) | 0.52 |
| Heart failure | ≥65 years | 1.16 (0.98–1.38) |  |

**Table S11 Medication-adjusted associations of FIB-4 with incident heart disease.**

| **Outcome** | **High vs low/indeterminate HR** | **Medications HR** | **Original model: per 1-unit HR** | **Medications HR** |
| --- | --- | --- | --- | --- |
| All heart disease | 1.18 (1.12–1.23) | 1.16 (1.11–1.22) | 1.07 (1.06–1.09) | 1.06 (1.05–1.08) |
| Coronary heart disease | 1.02 (0.95–1.10) | 1.01 (0.94–1.09) | 0.98 (0.96–1.005) | 0.98 (0.96–1.00) |
| Cardiac arrhythmias | 1.32 (1.24–1.41) | 1.29 (1.21–1.38) | 1.17 (1.14–1.19) | 1.15 (1.13–1.18) |
| Atrial fibrillation/flutter | 1.33 (1.24–1.43) | 1.30 (1.21–1.40) | 1.17 (1.15–1.20) | 1.16 (1.13–1.18) |
| Other cardiac arrhythmias | 1.26 (1.06–1.49) | 1.23 (1.04–1.46) | 1.12 (1.06–1.18) | 1.10 (1.04–1.16) |
| Heart failure | 1.29 (1.14–1.47) | 1.26 (1.11–1.44) | 1.13 (1.08–1.17) | 1.11 (1.07–1.16) |

**Table S12 Medication-adjusted associations of FIB-4 with CMR phenotypes.**

| **CMR phenotype** | **Original model: high vs low β/HR** | **Medications β/HR** | **Original model: per 1-unit β/HR** | **Medications β/HR** |
| --- | --- | --- | --- | --- |
| LV EDV† | 0.033 (0.018–0.047) | 0.031 (0.016–0.045) | 0.033 (0.029–0.037) | 0.032 (0.028–0.036) |
| LV ESV† | 0.044 (0.023–0.064) | 0.041 (0.020–0.061) | 0.040 (0.034–0.046) | 0.039 (0.033–0.045) |
| LV SV† | 0.024 (0.008–0.040) | 0.022 (0.006–0.038) | 0.028 (0.024–0.033) | 0.027 (0.023–0.032) |
| LV mass† | 0.014 (0.0003–0.027) | 0.013 (−0.0003–0.026) | 0.017 (0.013–0.020) | 0.016 (0.012–0.019) |
| LV EF | −0.473 (−0.975–0.028) | −0.451 (−0.951–0.049) | −0.276 (−0.420–−0.131) | −0.263 (−0.406–−0.120) |
| RV EDV† | 0.025 (0.010–0.039) | 0.023 (0.009–0.038) | 0.033 (0.029–0.037) | 0.032 (0.028–0.036) |
| RV ESV† | 0.022 (0.003–0.042) | 0.020 (0.001–0.039) | 0.036 (0.030–0.042) | 0.035 (0.029–0.041) |
| RV SV† | 0.026 (0.009–0.043) | 0.024 (0.007–0.041) | 0.031 (0.026–0.036) | 0.030 (0.025–0.035) |
| LA maximum volume† | 0.051 (0.025–0.077) | 0.049 (0.023–0.075) | 0.044 (0.036–0.051) | 0.043 (0.035–0.050) |
| LA minimum volume† | 0.083 (0.039–0.128) | 0.080 (0.036–0.124) | 0.069 (0.056–0.082) | 0.067 (0.054–0.080) |
| LA SV† | 0.038 (0.016–0.060) | 0.036 (0.014–0.058) | 0.030 (0.023–0.036) | 0.029 (0.022–0.035) |
| LA EF | −0.899 (−1.690–−0.108) | −0.861 (−1.651–−0.071) | −0.828 (−1.057–−0.599) | −0.801 (−1.029–−0.573) |
| RA maximum volume† | 0.021 (−0.003–0.046) | 0.020 (−0.004–0.044) | 0.046 (0.039–0.053) | 0.045 (0.038–0.052) |
| RA minimum volume† | 0.031 (−0.002–0.064) | 0.029 (−0.004–0.062) | 0.063 (0.053–0.072) | 0.061 (0.051–0.070) |
| RA SV† | 0.017 (−0.011–0.044) | 0.015 (−0.012–0.042) | 0.030 (0.022–0.038) | 0.029 (0.021–0.037) |
| RA EF | −0.285 (−1.064–0.495) | −0.273 (−1.051–0.505) | −0.766 (−0.991–−0.541) | −0.741 (−0.965–−0.517) |
| Concentric remodeling‡ | 2.12 (1.15–3.91) | 2.02 (1.09–3.75) | 1.12 (0.87–1.45) | 1.10 (0.85–1.42) |
| Eccentric hypertrophy‡ | 1.17 (0.43–3.16) | 1.13 (0.42–3.05) | 1.59 (1.36–1.86) | 1.55 (1.32–1.82) |
